# Trophic control changes with season and nutrient loading in lakes

**DOI:** 10.1111/ele.13532

**Published:** 2020-05-31

**Authors:** Tanya L. Rogers, Stephan B. Munch, Simon D. Stewart, Eric P. Palkovacs, Alfredo Giron‐Nava, Shin‐ichiro S. Matsuzaki, Celia C. Symons

**Affiliations:** ^1^ Southwest Fisheries Science Center National Marine Fisheries Service National Oceanic and Atmospheric Administration Santa Cruz CA 95060 USA; ^2^ Cawthron Institute Nelson New Zealand; ^3^ Department of Ecology and Evolutionary Biology University of California Santa Cruz, Santa Cruz CA 95060 USA; ^4^ National Center for Ecological Analysis and Synthesis University of California Santa Barbara, Santa Barbara CA 93101 USA; ^5^ Center for Environmental Biology and Ecosystem Studies National Institute for Environmental Studies 16‐2 Onogawa Tsukuba Ibaraki 305‐8506 Japan; ^6^ Department of Ecology and Evolutionary Biology University of California Irvine, Irvine CA 92697 USA

**Keywords:** consumer control, empirical dynamic modelling, nutrients, resource control, species interactions, temperature, time series

## Abstract

Experiments have revealed much about top‐down and bottom‐up control in ecosystems, but manipulative experiments are limited in spatial and temporal scale. To obtain a more nuanced understanding of trophic control over large scales, we explored long‐term time‐series data from 13 globally distributed lakes and used empirical dynamic modelling to quantify interaction strengths between zooplankton and phytoplankton over time within and across lakes. Across all lakes, top‐down effects were associated with nutrients, switching from negative in mesotrophic lakes to positive in oligotrophic lakes. This result suggests that zooplankton nutrient recycling exceeds grazing pressure in nutrient‐limited systems. Within individual lakes, results were consistent with a ‘seasonal reset’ hypothesis in which top‐down and bottom‐up interactions varied seasonally and were both strongest at the beginning of the growing season. Thus, trophic control is not static, but varies with abiotic conditions – dynamics that only become evident when observing changes over large spatial and temporal scales.

## Graphical abstract

We estimated time‐varying trophic interaction strength between zooplankton and phytoplankton in 13 globally‐distributed lakes over decadal timescales using empirical dynamic modelling. Trophic interactions were not static, but varied seasonally (within lakes) and with abiotic conditions (across lakes) ‐ nonlinear dynamics which are only evident when pairing long‐term observational data with cutting edge modelling techniques.

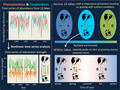

‘Clear cause and effect relationships do not emerge from multiple regression analyses of lake ecosystem data. Experimental manipulations of food webs are a more promising research strategy’ – Carpenter et al. 1985

## Introduction

The extent to which biomass in food webs is controlled by resource supply (bottom‐up effects) or limited by higher trophic levels (top‐down effects) has been a central question in ecology for almost a hundred years (Elton 1927; Lindeman 1942; Hairston *et al.* 1960; Polis 1999). After Carpenter & colleagues (1985) described the difficulty of inferring causal relationships in lake ecosystems from observational data, a tradition of whole‐ecosystem manipulation in limnology began. Indeed, much of our understanding of variation in trophic control has come from manipulative or ‘natural’ experiments where consumers have been excluded or introduced, or basal resource availability has been altered (Borer *et al.* 2005). Experiments such as these can provide information about the abiotic and biotic conditions that mediate the strength of top‐down (TD) or bottom‐up (BU) effects. Knowledge of these drivers can help us predict when changing environmental conditions will have cascading impacts throughout food webs (Chamberlain *et al.* 2014).

Although experimental approaches remain the gold standard for inferring mechanisms in ecology, experiments have limitations. Because of the short timescales, logistical and analytic limitations, and the rarity with which studies are repeated (but see Power *et al.* 2008; Barton & Schmitz 2009; Pace *et al.* 2019), the extent to which trophic control varies through time is poorly described (McMeans *et al.* 2015; Piovia‐Scott *et al.* 2017). In addition, short‐term experiments are inherently transient (Hastings 2004) and necessarily ignore ecological and evolutionary processes occurring over longer timescales, such as turnover in species composition and local adaptation (Siepielski *et al.* 2009). The limited spatial extent of experiments is also problematic: While controlled whole‐ecosystem manipulations may be tractable in small, replicated systems (e.g. small lakes, islands), whole‐ecosystem experiments are intractable for large, unreplicated ecosystems (e.g. large lakes, ocean basins). Finally, many experimental manipulations are extreme (e.g. complete predator removal), do not always reflect natural conditions and rates of biotic or abiotic change, and often have important ethical considerations.

These challenges suggest an important role for observational data for gaining insight into spatio‐temporal variation in trophic control. Monitoring data encompass much larger spatio‐temporal scales than experiments and capture environmental fluctuations across which trophic interaction strength might vary (Piovia‐Scott *et al.* 2017). However, previous attempts to quantify BU/TD control from observational data have used linear correlations or regressions, and assume that negative and positive relationships between adjacent trophic levels respectively demonstrate TD and BU control (Jeppesen *et al.* 2003; Bunnell *et al.* 2014; Boyce *et al.* 2015). These linear models provide only a single, static estimate of interaction strength; hence no information can be gleaned about temporal variation in interaction strengths, which may result from seasonality, context‐dependency or nonlinearity. For example herbivores can decrease algal biomass through grazing (Carpenter & Kitchell 1988) or increase algal biomass through nutrient recycling (e.g. Attayde & Hansson 1999; Herren *et al.* 2017). Yet, when and where these alternative outcomes occur in natural lakes is not known. Indeed, the sign of correlations between nonlinearly coupled variables can change over time, even with no change in underlying dynamics or external forcing (Sugihara *et al.* 2012). Finally, empirical estimates of BU and TD effects often fail to recognise that both effects occur simultaneously.

There are three main ways we might expect abiotic variables to influence trophic control. First, nutrient concentration and stoichiometry may influence BU control through nutrient limitation of primary producers (Elser *et al.* 2000, 2007; Rosenblatt *et al.* 2016). Nutrients may also alter TD control from herbivores by altering resource quantity and quality (Leibold 1989; Polis 1999). Second, temperature may alter trophic interaction strengths through effects on biological rates, as summarised by the Metabolic Theory of Ecology (MTE; Brown *et al.* 2004). In general, MTE predicts greater consumer control at warmer temperatures due to the differential responses of autotrophic and heterotrophic metabolism to warming (Lopez‐Urrutia *et al.* 2006; O’Connor *et al.* 2011). Finally, ‘resets’ that occur seasonally or after a disturbance may influence the temporal dynamics of TD and BU control by preventing settlement to equilibrium (Hutchinson 1961; McCann *et al.* 1998). For example early successional communities should favour rapidly growing prey, with TD effects decreasing over time as persistent consumption selects for better‐defended and inedible prey (Piovia‐Scott *et al.* 2017). The structure of food webs can also influence trophic control. For example species diversity may increase BU and decrease TD control through complementarity (Pimm 1982; Polis 1999; Chase *et al.* 2000; Finke & Denno 2004). Lastly, we might expect herbivores and predators to show opposite‐signed relationships with primary producers, typical of a trophic cascade (Estes et al. 1995).

New methodologies for nonlinear time series analysis which can estimate the strength of BU and TD control at every timepoint, show promise for more nuanced inference of trophic interactions from observational data. These methods, collectively known as empirical dynamic modelling (EDM), employ nonparametric regression techniques which do not assume species interactions are constant, and produce time series of interaction strengths with varying sign and magnitude (Deyle *et al.* 2016). Hence, ‘bottom‐up control’ and ‘top‐down control’ become quantitative, continuous variables. EDM also incorporates time lags of observed variables to account for delayed effects and compensate for unobserved variables (Ye *et al.* 2015; Munch *et al.* 2019). EDM has recently been used to explore causal links between grazers, environmental drivers and phytoplankton in Lake Kasumigaura (Matsuzaki *et al.* 2018) and Lake Geneva (Anneville *et al.* 2019), but these studies did not examine changes in species interactions over time. These methods have not yet been used across multiple systems encompassing a wide range of environmental conditions.

**Figure 1:**
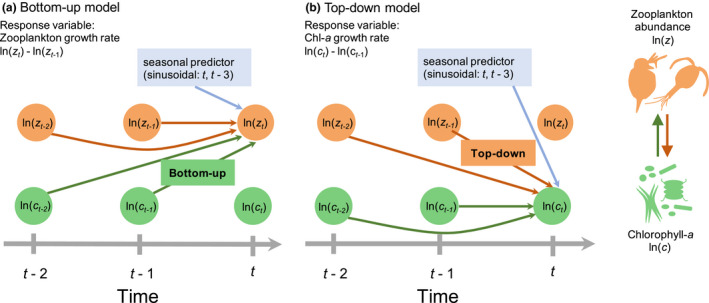
Diagram of model structure for (a) zooplankton and (b) chl‐a growth rates.

In this study, we use EDM to examine how the strength of TD and BU control shifts temporally in response to the environment using observational data from multiple lakes from around the world. Specifically, we quantify variation in trophic interactions between phytoplankton (chlorophyll‐a) and zooplankton. We compiled time series data from 13 lakes spanning a wide range of nutrient conditions (oligotrophic to eutrophic) and for each lake, fit two models to quantify BU and TD interactions. In the BU model, we examine the effect of past chlorophyll‐a concentration on zooplankton population growth rate as our measure of BU control, and in the TD model, we examine the effect of past zooplankton abundance on chlorophyll‐a growth rate as our measure of TD control (Fig. 1). We address four main questions: (1) How do BU and TD interaction strengths vary over time in natural systems? (2) What abiotic and biotic drivers are associated with variation in TD and BU control? (3) Do the relevant drivers differ across vs. within lakes? and (4) Do trophic interaction strengths differ between herbivorous and predatory zooplankton? Using these methods, we are able to explore whether trophic interaction strengths, assumed constant in other studies, are temporally variable, and whether this variability aligns with theoretical expectations across larger scales of space and time than can be captured in short‐term experiments.

## Methods

### Data

**Figure 2:**
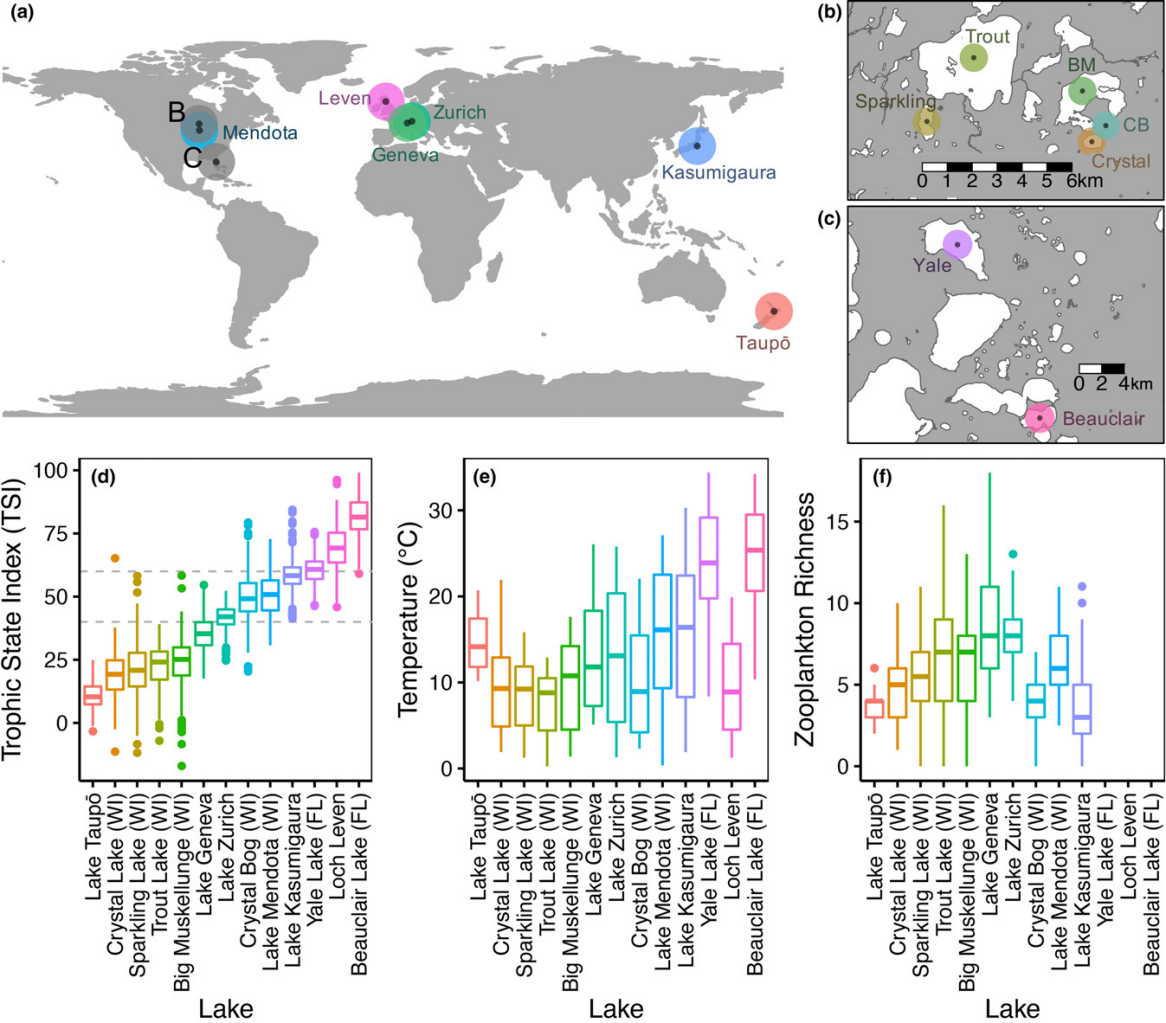
The (a–c) geographic location, (d) trophic state index (TSI), (e) water temperature, and (f) zooplankton species richness of the 13 lakes used in this study. Sites are ordered here (and in all other figures) by median TSI. Lakes with TSI < 40 are considered oligotrophic, lakes with TSI> 60 are considered eutrophic and lakes with intermediate values are considered mesotrophic (values denoted by dashed horizontal lines). WI = lakes in Wisconsin that are part of the North Temperate Lakes Long Term Ecological Research region, FL = lakes in Florida. In (b), BM = Big Muskellunge, CB = Crystal Bog.

We compiled data on zooplankton abundance, chlorophyll‐a concentration (chl‐a), water chemistry (e.g. nutrient concentrations), temperature and lake size from 13 globally distributed lakes (Fig. 2a–c, Table S1; Gunn *et al.* 2015; National Institute for Environmental Studies 2016; Takamura *et al.* 2017; Magnuson *et al.* 2019; Magnuson *et al.* 2020). We only included lakes with monthly or nearly monthly sampling of all variables (if multiple observations were taken during a month, these were averaged) and that had at least 75 usable time points for zooplankton and chl‐a. If measurements of chl‐a, water chemistry and temperature (Fig. 2e, Fig. S1) were taken at multiple depths, we averaged measurements taken at depth ≤ 2 m.

For each lake, we calculated zooplankton abundance as the sum of all herbivorous copepod and cladoceran densities at each time point (Table S2). Zooplankton biomass was used when available (Lakes Yale and Beauclair), and models with either metric produced qualitatively similar results (Fig. S2). Rotifers were excluded because they were not enumerated for many of the lakes in the data set. We also calculated zooplankton species richness as the number of herbivorous copepod and cladoceran species observed at each time point (Fig. 2f). Genera not identified to species were counted as a species, but we did not count unidentified nauplii and copepodites. Separately, we also calculated the total abundance (density) of predatory zooplankton at each time point.

We used chl‐a at each time point as a proxy for overall phytoplankton biomass. If a measurement of chl‐a was below the detection threshold, it was replaced with half the minimum recorded value of chl‐a in that lake.

The lakes in our analysis spanned a wide range of nutrient conditions with chl‐a, total nitrogen (TN) and total phosphorus (TP) spanning roughly three orders of magnitude (Fig. S1). To provide a measure of eutrophication (nutrient conditions) for each timepoint in each lake, we calculated the trophic state index (TSI) according to Paulic *et al. *(1996) using the measurements of chl‐a, TN and TP (Fig. 2d). Lakes with TSI < 40 are considered oligotrophic, lakes with TSI> 60 are considered eutrophic and lakes with intermediate values are considered mesotrophic.

### Analysis

We used s‐map EDM (Sugihara *et al.* 1994) to model population growth as a function of past population abundance and relevant covariates as implemented in rEDM (Ye *et al*. 2018). S‐map performs locally weighted multiple linear regression at each timepoint (Sugihara *et al.* 1994). The parameter θ controls the amount of local weighting, where a value of 0 corresponds to a global linear model (equivalent to a vector autoregressive model; Deyle *et al.* 2016) and higher values correspond to greater amounts of local weighting (greater nonlinearity). Importantly, the local weighting is based on closeness in predictor space, not closeness in time, such that relationships are determined not by what happened most recently, but by what happened when conditions were similar. If the covariates include abundances of other species, the local slope coefficients (partial derivatives) associated with those species provide an estimate of interspecific interaction strength at each point in the time series (Deyle *et al.* 2016).

In our analysis, we fit two models for each lake (Fig. 1):

$$\text{Bottom}-\text{up model}:\;z_{t}-z_{t-1}\;=\;f\left(z_{t-1},z_{t-2},c_{t-1},c_{t-2},s_{t},s_{t-3}\right)$$

$$\text{Top}-\text{down model}:\;c_{t}-c_{t-1}\;=\;g\left(z_{t-1},z_{t-2},c_{t-1},c_{t-2},s_{t},s_{t-3}\right)$$

where $z_{t}$ is the natural log of zooplankton abundance scaled to mean 0 and variance 1, $c_{t}$ is the natural log of chl‐a scaled to mean 0 and variance 1, and $z_{t}-z_{t-1}$ and $c_{t}-c_{t-1}$ are scaled zooplankton and chl‐a growth rates, respectively, on a monthly time step. To control for seasonality, we included the ‘seasonal predictors’ $s_{t}$ and $s_{t-3}$, which were sine functions (mean 0, variance 1) with a period 12 months, offset by a quarter period. To select θ (the local weighting parameter), we fit each model using a range of different values (0–8), and selected the value that minimised mean squared error (maximised *R* ^2^) using leave‐one‐out cross‐validation. Output from the s‐map model includes coefficients associated with each predictor at each timepoint. We fit models separately for herbivorous and predatory zooplankton. The following analyses were performed only for herbivorous zooplankton, as predatory zooplankton were only encountered frequently enough to analyse (> 60% zeros) in three of the 13 lakes.

For each lake, we first calculated the proportional influence of zooplankton, chl‐a, and seasonality on zooplankton and chl‐a growth rates. For each time point, we divided the summed squared coefficients associated with zooplankton, chl‐a and seasonality (both lags), by the summed squared coefficients for all predictors, and then averaged these proportions across all timepoints. This calculation reflects the proportion of the explained variance in growth rate that is attributable to each set of predictors.

We next focused on the coefficients associated with the cross‐trophic level predictors $\delta f/\delta c_{t-1}$ (the effect of chl‐a at *t*‐1, $c_{t-1}$, on subsequent zooplankton growth rate, $z_{t}-z_{t-1}$, in the BU model, f) and $\delta g/\delta z_{t-1}$ (the effect of zooplankton at *t*‐1, $z_{t-1}$, on subsequent chl‐*a* growth rate, $c_{t}-c_{t-1}$, in the TD model, g). These coefficients provide the estimates of BU and TD interaction strength, respectively, at each timepoint, for each lake. Since zooplankton consume phytoplankton, we expected the TD coefficients to be negative and the BU coefficients to be positive, with values farther from zero indicating stronger effects, and values close to zero indicating weak or no effect. However, since the monthly timestep integrates both direct and indirect effects, signs opposite of this expectation are possible. Accumulation of indirect effects also makes it difficult to interpret coefficients at lags longer than 1, so we focus only on those at time *t*‐1.

We examined the extent to which different abiotic and biotic variables and seasonality could explain variation in BU and TD interaction strengths both across and within lakes. To accomplish this, we fit regression models to the BU and TD coefficients. To evaluate relationships across the full range of conditions experienced *across* lakes, we combined data and coefficients across lakes and regressed interaction strengths against the two seasonal predictors ( $s_{t}$ and $s_{t-3}$), TSI, temperature, and species richness. TSI, temperature, and species richness were included as 2nd order orthogonal polynomials to allow for linear, curvilinear or unimodal relationships, which are what we might reasonably hypothesise based on existing literature. Since the three highest nutrient lakes lacked species richness data and excluding these lakes might have biased the results with respect to nutrients, we evaluated the effect of species richness (for the sites that had these data) on the residuals from a model containing all of the other predictors based on data from all lakes. Seasonal predictors for the one southern hemisphere lake (Lake Taupō, New Zealand) were shifted by 6 months so as to seasonally align with the northern hemisphere lakes. To evaluate relationships *within* a given lake (which could differ from the across‐lake response and vary among lakes), we fit regression models separately for each individual lake. Because the seasonal predictors and temperature were strongly collinear within lakes, we used one linear model to quantify the proportion of variation in interaction strength due to general seasonality (the two seasonal predictors), and a separate second‐order polynomial model to evaluate the variation due to TSI, temperature, zooplankton abundance, zooplankton species richness (if available). Zooplankton abundance was not used in the across‐lake model because units could not be standardised across lakes. For both the across‐ and within‐lake analysis, we fit separate models for the BU and TD coefficients. Timepoints with measurements of chl‐a, TN or TP below the detection threshold were excluded. We calculated effect sizes for each predictor as the proportion of variation explained by that predictor after accounting for the variance explained by the other predictors in the model (partial η^2^, Cohen 1973).

Conditional responses to predictors were calculated using the ‘effects’ package (Fox & Weisberg, 2018, 2019). All analyses were performed in R v.3.6.1 (R Core Team 2016).

## Results

The BU and TD models were able to explain a large proportion of the variation in zooplankton and chl‐a growth rates respectively (leave‐one‐out *R* ^2^ values ranging from 0.23 to 0.70), and their dynamics were mostly nonlinear (θ > 0 for many lakes; Table S3). Our initial analysis included 20 lakes, but in seven of these data sets, either zooplankton or chl‐a growth rate was not predictable from the fitted models (leave‐one‐out *R* ^2^ < 0.20), and they were removed from further analysis, leaving us with 13 lakes (Fig. 2). Removed lakes included two lakes in the North Temperate Lakes Long Term Ecological Research region (Fig. 2b; Table S3) and five in the Florida region (Fig. 2c; Table S3).

### Variation in trophic control across lakes

Of the explainable variation in growth rate, the proportion attributable to within‐trophic level effects (e.g. past values of chl‐a for chl‐a growth, indicative of density‐dependence) tended to be the largest (Fig. S3). For chl‐a growth, the proportion attributable to across‐trophic level effects (TD effects), which ranged from 0.007 to 0.35, was greatest at mid‐high nutrient levels. For zooplankton growth, the proportion attributable to across‐trophic level BU effects did not show a pattern with respect to nutrients. The proportion attributable to seasonality, which ranged from 0.06 to 0.50, decreased with increasing nutrient levels for chl‐a growth, and was greatest at intermediate nutrient levels for zooplankton growth.

**Figure 3:**
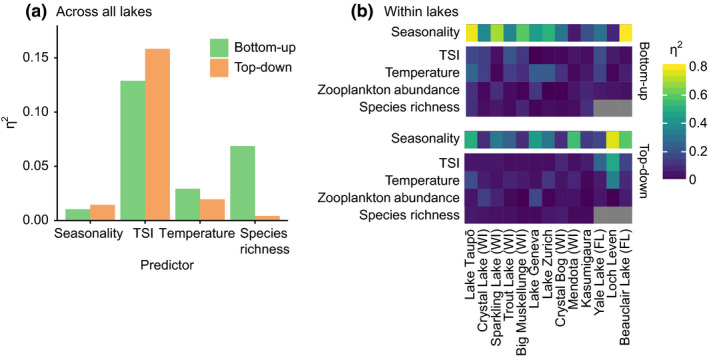
Proportion of variation in BU and TD interaction strength coefficients for herbivorous zooplankton attributable to different predictors (a) across all lakes, and (b) within each lake. Seasonality includes the effect of both sinusoidal predictors. Values reflect the proportion of the total variance in interaction strength attributable to each predictor, after accounting for the variance explained by the other predictors. Within lakes, variation due to seasonality was evaluated in a separate regression model because of collinearity with temperature.

**Figure 4:**
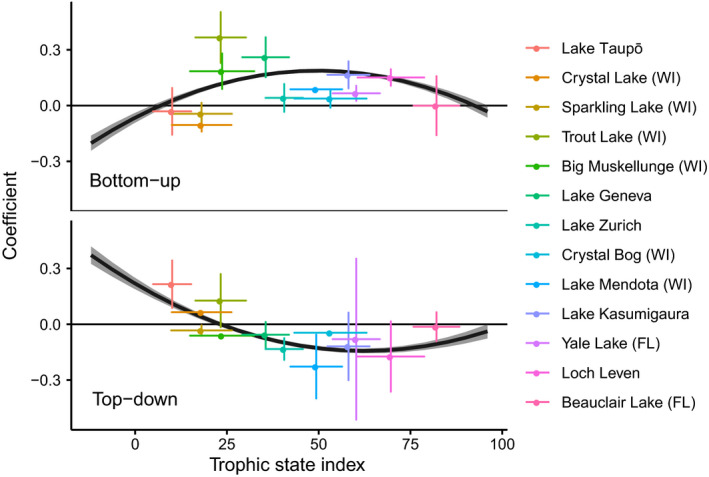
Conditional effects of trophic state index (TSI) on BU and TD interaction strength coefficients for herbivorous zooplankton, evaluated across lakes. Curves are fit to all data points; to reduce clutter, only means and standard deviations of TSI and coefficients are shown. All data points are shown in Fig. S4.

Of the four predictors used in the across‐lake regression model, TSI explained the most variation in both TD and BU effects, and explained more variation in TD than BU effects (Fig. 3a). The effect of TSI on BU interaction strength showed a hump‐shaped relationship, with most positive values at intermediate TSI (Fig. 4). Conversely, the effect of TSI on TD interaction strength was u‐shaped, with positive values at low TSI, negative values at intermediate to high TSI and values close to zero at the highest TSI levels (Fig. 4). The effects of temperature and species richness, although also statistically significant, explained relatively little variation in BU or TD interaction strength (Table S4, Fig. S4). Both BU and TD effects were stronger (farther from zero) at lower temperatures and weaker (closer to zero) at higher temperatures. Residuals from the across‐lake model (both BU and TD) did not show any obvious relationship with lake surface area **(**Fig. S5).

### Variation in trophic control within individual lakes

**Figure 5:**
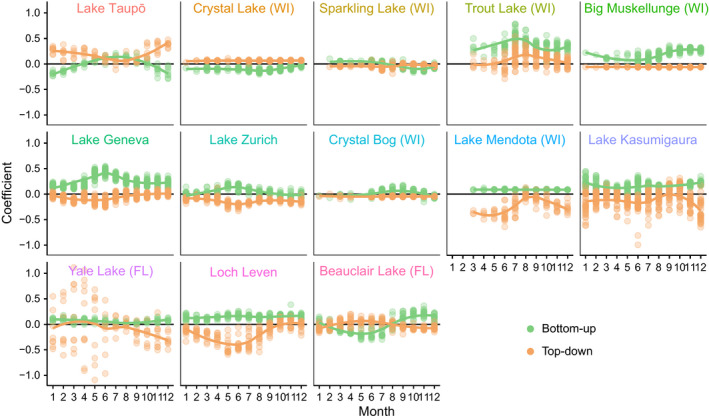
Seasonal variation in BU and TD interaction strength coefficients within lakes. Curves are splines.

Within most lakes, seasonality was apparent in both the BU and TD interaction coefficients (Fig. 5; Table S5). On average, a seasonal sinusoidal function could account for 40% and 24% of the variation in BU and TD interaction strengths respectively (Fig. 3b). BU and TD interaction strengths tended to be of greatest magnitude around the same time of the year, and peaked during the spring in many lakes (Fig. 5). For the few lakes for which no seasonality was apparent (5 for TD, 2 for BU), four had linear or near‐linear dynamics (θ ≤ 0.3) and hence constant interaction strength, and three had nonlinear dynamics (θ ≥ 1.5) (Table S3).

In contrast to the across‐lake analysis, very little of the within‐lake variation in BU and TD interaction strengths could be accounted for by the four abiotic and biotic variables (Fig. 3b **;** Table S6). Which of these variables had the largest effect size was also very inconsistent among lakes. For TD effects, TSI explained the most variation in the three highest nutrient lakes (Fig. 3b).

### Herbivorous and predatory zooplankton

**Figure 6:**
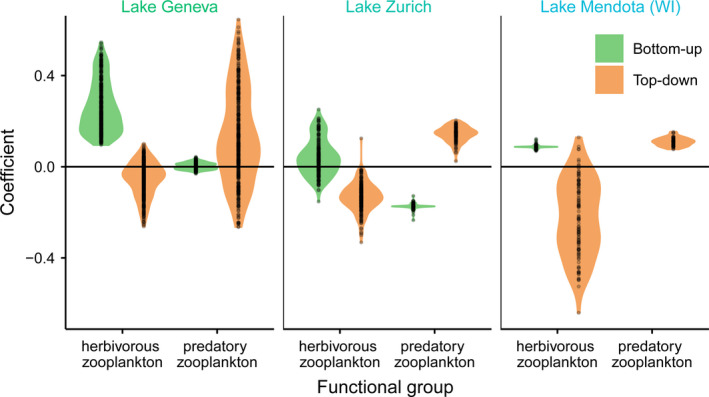
Distribution of BU and TD interaction strength coefficients for herbivorous zooplankton (copepods and cladocerans) and predatory zooplankton in Lake Geneva, Lake Zurich and Lake Mendota. Values for BU effects of predatory zooplankton in Lake Mendota not shown due to poor model fit (*R* ^2^ < 0.2).

Predatory zooplankton was enumerated and encountered frequently enough to analyse in Lake Zurich, Lake Geneva and Lake Mendota, three lakes with intermediate nutrient levels. In contrast to herbivorous zooplankton, which has negative TD effects in these lakes, predatory zooplankton had positive TD effects on chl‐a growth rates, meaning that high past abundances of predators increased chl‐a concentrations, possibly through cascading trophic interactions (Fig. 6). Coefficient distributions for models fit to different herbivorous zooplankton functional groups (copepods, small cladocerans and large cladocerans) are shown in Fig. S6. Results for copepods and large cladocerans tended to reflect the results for total herbivorous zooplankton, whereas the results for small cladocerans were more idiosyncratic. The species included in each functional group are given in Table S2.

## Discussion

Because of the difficulties of inferring ecological interactions from observational data, ecologists have relied upon manipulative experiments to test the relative strength of TD and BU effects in ecosystems (Paine 1974; Schindler 1977; Carpenter *et al.* 1985; Power *et al.* 2008; Estes *et al.* 2011; Pace *et al.* 2019). However, even the largest scale experiments involving whole‐ecosystem manipulations of lakes use relatively small lakes and run over one or just a few years, which may not scale to larger lakes or longer timescales (Schindler 1998). Nonlinear time series analysis allows us to begin unravelling the complexity of bidirectional TD and BU interactions using observational data collected over long time scales in real‐world ecosystems. Using time series data from 13 lakes from around the world, we show that interaction strength between adjacent trophic levels is highly dynamic, and this variation displays relationships with productivity and seasonality.

### Variation in trophic control across lakes

Across all lakes, the TD effect of zooplankton on phytoplankton displayed a clear relationship with nutrient conditions (summarised by TSI; Figs 3 and 4). In the most oligotrophic lakes (chl‐a < 2 µg L^‐1^), TD coefficients were positive, suggesting that zooplankton promote phytoplankton growth. A possible explanation is that excretion by herbivorous zooplankton is an important source of recycled nutrients for phytoplankton (Brabrand *et al.* 1990; Vanni 2002; Shostell & Bukaveckas 2004), and that in these nutrient‐limited lakes, the resultant increase in producer growth rate exceeds mortality due to herbivory (Sterner 1986). In contrast, under medium and high nutrient levels, TD coefficients were negative, as predicted for TD control (phytoplankton suppression) due to herbivory. Moderate resource availability and quality is expected to increase consumption rates by herbivores, thereby increasing the impacts of primary consumers on primary producers (Leibold 1989; Polis 1999). Finally, in the most eutrophic lakes, TD coefficients were around zero. At very high concentrations of phytoplankton, grazing may have a negligible impact on phytoplankton dynamics. Another possible explanation is that highly eutrophic lakes are often dominated by inedible and toxic phytoplankton species such as cyanobacteria (Moustaka‐Gouni *et al.* 2006), and while adaptation can ameliorate the fitness effects of cyanotoxins (Sarnelle & Wilson 2005), most zooplankton are not able to ingest enough cyanobacteria to control their abundance (Tillmanns *et al.* 2008).

According to these findings, nutrient addition to oligotrophic lakes may increase TD grazing pressure, whereas nutrient addition to more eutrophic systems may have the opposite effect. This nonlinear relationship between TD control and nutrient loading may have obscured the role of nutrients in past studies of trophic coupling. For example a meta‐analysis of trophic cascade strength found a trend for stronger trophic cascades when nutrients were elevated, although this effect was not significant (Borer *et al.* 2005). Quantifying the shape of these nonlinear relationships may elucidate important interactions between abiotic conditions and trophic control and is an important consideration when setting nutrient limits for lakes to maintain ecological states. Other approaches which assume either BU and TD control from global positive and negative correlations may misclassify positive TD effects as BU effects, leading to an underestimation of total coupling within the ecosystem.

### Variation in trophic control within individual lakes

Within individual lakes, trophic control displayed seasonal fluctuations (Figs 3b and 5). However, this seasonality was not strongly or consistently associated with seasonality in any abiotic or biotic variables considered, including TSI. This may be due to the presence of multiple underlying environmental drivers, and different drivers being important at different times of the year. For example light and nutrients may be more important at the beginning of the growing season and temperature at the end (Sommer *et al.* 1986). Another possibility is that the relationship between TSI and trophic control may only be apparent across larger ranges of variation than are experienced within individual lakes. Shared responses within and among lakes may not be detectable because, as is found with other ecosystem properties (Sorrano et al. 2019), temporal variation in TSI within any single lake was much smaller than spatial variation across all lakes (Fig. 4). Additionally, strong within‐lake coupling between food web dynamics and nutrient availability that is itself dependent on overall nutrient levels may obscure relationships between nutrients and interaction strength.

Many lakes displayed an increase in TD and BU control at the beginning of the growing season that declined as the season progressed (Fig. 5). For the one Southern hemisphere lake, Lake Taupō, this peak occurred during the austral growing season. An initial increase in both BU and TD control at the beginning of the growing season is predicted by theory on trophic interactions, disturbance and successional patterns in seasonal systems, which is consistent with the classic PEG (Plankton Ecology Group) model in limnology (Sommer *et al.* 1986). BU and TD control are expected to be a transient phenomenon, with conditions at the beginning of the growing season favouring rapidly growing primary producers (high BU control; Sommer *et al.* 1986). As the season progresses, TD grazing selects for better defended algal species (Holt *et al.* 1994; Agrawal 1998), induces defences (Van der Stap *et al.* 2007), or causes adaptation (Ingram *et al.* 2012; Schaffner *et al.* 2019) that reduces TD control. At the end of the growing season, unfavourable environmental conditions will then ‘reset’ the system. The fact that seasonal maxima in both BU and TD control often occurred around the same time of year further demonstrates that systems should not be dichotomously grouped as BU‐ or TD‐controlled, as is often done when using correlation‐based analyses.

Our results highlight the importance of seasonality as a driver of biological interactions – results which might be missed in studies which ‘remove’ seasonality from data prior to analysis. They also highlight the potential sensitivity of trophic control to changes in seasonality in lake ecosystems. Climate change impacts on seasonality are known to alter ecosystems by extending or restricting periods of specific community interactions (Winder & Schindler 2004; de Sassi & Tylianakis 2012). In lakes, climate change is expected to increase mean water temperatures, the length of the ice‐free season and the strength and duration of thermal stratification. These changes can have varied effects. As temperatures have warmed, lakes have experienced earlier spring phytoplankton blooms followed by an increase in *Daphnia* abundance which leads to a long‐lasting clear‐water phase (Adrian *et al.* 2016). Simultaneously, the longer stratified period reduces nutrient availability deep in the water column where light is limiting, favouring phytoplankton species less vulnerable to zooplankton grazing (Anneville *et al.* 2002). Warming also increases the dominance of cyanobacteria particularly in nutrient‐rich lakes (Paerl & Huisman 2008). In eutrophic lakes, the warmer, longer growing season may reduce the ability of zooplankton to control algal blooms, especially as the period of low TD control lengthens (Fig. 5), decreasing coupling between phytoplankton and herbivores. By contrast, longer summer stratification in oligotrophic lakes will extend periods of nutrient depletion (Verburg *et al.* 2003) and may strengthen interactions between phytoplankton and herbivores as nutrient recycling becomes more important (Fig. 4).

### Variables not related to trophic control

While our findings were consistent with some existing hypotheses (e.g. seasonal resets and nutrient recycling), other results were more surprising. For instance the balance between TD and BU forces is expected to respond to temperature because different organisms and physiological processes vary in their thermal sensitivity (Allen *et al.* 2005; Dell *et al.* 2014). Mathematical models using the Metabolic Theory of Ecology (e.g. Vasseur & McCann 2005) and experiments (e.g. Shurin *et al.* 2012) indicate that TD control increases at higher temperatures. However, we found that temperature alone did not explain much variation in trophic control across lakes or have a consistent effect within lakes (Fig. 3). This may be due to turnover in traits, species and functional groups over time and space that is ubiquitous in real ecosystems but neglected in typical models and experiments. Light and stratification may also influence plankton dynamics more than temperature (Sommer *et al*. 2012).

Despite a large body of theory on why BU and TD control should vary with species richness (Pimm 1982; Polis 1999; Chase *et al.* 2000; Finke & Denno 2004), we did not find a relationship (Fig. 3). Meta‐analyses of experiments conducted in aquatic and terrestrial systems have similarly concluded that there is no general relationship between species diversity and TD control (Borer *et al.* 2005; O’Connor & Bruno 2009). It is possible that trait distributions are more important than the number of species, *per se*, causing species richness to be too coarse a metric to predict the strength of trophic interactions in food webs.

### Herbivorous and predatory zooplankton

For the three lakes in which we were able to examine predatory zooplankton, past predatory zooplankton abundance increased chl‐a growth rate (Fig. 6). This result is consistent with a trophic cascade (i.e. an indirect positive effect on primary producers resulting from the consumption of herbivores). Invertebrate predators are known to have cascading impacts on phytoplankton in lakes (e.g. Walsh *et al.* 2016). Thus, the different patterns of TD interaction strengths for herbivores and predators highlight the ability of EDM to recapitulate species interactions in nature solely from observational data. Future studies might further explore interactions among different zooplankton and phytoplankton functional groups, including rotifers, which may be numerically dominant in eutrophic lakes (Matsuzaki *et al.* 2018). Studies might also explore data sets with information on the traits of individuals, which may be an important determinant of interaction strengths (Shurin *et al.* 2002, 2006).

Most evidence of lake trophic cascades comes from the manipulation of fish assemblages (Estes 1995). All analysed lakes contain fish, and fish are likely driving some of the dynamics in zooplankton and chl‐a over time (e.g. Liu *et al.* 2019). Since we lacked monthly fish data, we were unable to include fish in the model directly; however, seasonality in fish predation rates (e.g. due to seasonal peaks in the production of planktivorous fish larvae) would ideally be captured by the generic ‘seasonal predictors’. This may explain why apparent seasonality in trophic interaction strength does not depend directly on temperature.

## Conclusions

Our results capture the tight BU and TD coupling between primary producers and herbivores, and allow us to explore factors related to spatio‐temporal variation in this coupling. These findings emerged entirely from the dynamics in observational data, highlighting the ability of monitoring data to capture ecological processes occurring over large scales of space and time. Carpenter and colleagues correctly asserted that regression analyses could not adequately capture trophic dynamics because of transient dynamics and time lags; however, experimental approaches are no longer alone in their ability to capture these complexities. While we clearly still need experiments and natural history studies to understand *mechanisms*, modern dynamical systems approaches which allow for state‐dependent coefficients, time lags, and nonlinearity make it possible to estimate species interaction strengths from observational data and test hypotheses over spatio‐temporal scales that would be difficult or impossible to otherwise. Ideally, analyses like these will allow us to better anticipate the effects of nutrient loading, seasonality, and other variables on lake trophic dynamics, and in the many other ecosystems where these methods could be applied.

## Authorship

TLR and CCS conceived the project idea. All authors contributed to the design of the project. CCS, TLR, SDS, SSM and AG collated data. TLR performed the analyses with input from SBM. TLR and CCS created the figures and led the writing. All authors contributed to the editing of the manuscript.

## Supporting information

Supplementary Material

## Data Availability

No new data were used, and data sources can be found in Table S1.
