# Supplementary material for: Trophic control changes with season and nutrient loading in lakes
Source: Ecol Lett. 2020 May 31;23(8):1287–97. doi: 10.1111/ele.13532 (PMC7384198; doi:10.1111/ele.13532)
Supplement: Supplementary file 1 — Supplementary Material [file ELE-23-1287-s001.pdf]

SUPPLEMENTARY MATERIAL

Table S1. Metadata for lake time series used in analysis. Trophic levels were calculated considering producers as trophic level 1.

Lake Name	Country	Latitude	Longitude	Surface Area (km^2)	Depth (m) Zmax [mean]	Thermal regime (duration of ice cover)	Trophic Levels (apex predators)	Start-End Year	Data Source
Lake Taupō	New Zealand	-38.797110	175.896818	616	186 [110]	Warm monomictic (ice-free)	3-4 (Rainbow trout, Brown trout)	2000-2009	https://bps.waikatoregion.govt.nz/online-services/new/requestforservice/step/1
Crystal Lake	WI, United States	46.0018	-89.6136	0.375	20.4 [10.4]	Dimictic (138 days)	4 (Lake Trout, Largemouth Bass, Northern Pike)	1981-2017	https://lter.limnology.wisc.edu/data
Sparkling Lake	WI, United States	46.0091	-89.6995	0.637	20 [10.9]	Dimictic (136 days)	4 (Walleye and Largemouth Bass)	1981-2017	https://lter.limnology.wisc.edu/data
Big Muskellunge	WI, United States	46.0162	-89.6135	3.6	21.3 [7.9]	Dimictic (141 days)	4 (Northern Pike and Walleye)	1981-2017	https://lter.limnology.wisc.edu/data
Trout Lake	WI, United States	46.0461	-89.6751	16.1	35.7 [14.6]	Dimictic (135 days)	4 (Northern Pike, Lake Trout and Walleye)	1981-2017	https://lter.limnology.wisc.edu/data
Lake Geneva	Switzerland /France	46.433333	6.55	580	310 [154]	Warm monomictic (ice-free)	4 (Pike)	1959-2018	© SOERE OLA-IS, AnaEE-France,INRA of Thonon-les-Bains, CIPEL, Dec 19 2019, developed by the Eco-Informatique ORE system of the INRA
Lake Zurich	Switzerland	47.235466	8.698674	88.7	136 [49]	Warm monomictic (ice-free)	4 (Pike)	1987-2006	Wasserversorgung Zurich
Lake Mendota	WI, United States	43.1113	-89.4255	39.4	25 [12.8]	Dimictic (119 days)	4 (Northern Pike and Walleye)	1976-2018	https://lter.limnology.wisc.edu/data

Lake Name	Country	Latitude	Longitude	Surface Area (km^2)	Depth (m) Zmax [mean]	Thermal regime (duration of ice cover)	Trophic Levels (apex predators)	Start-End Year	Data Source
Crystal Bog	WI, United States	46.0076	-89.6063	.006	2.5 [1.7]	Polymictic (138 days)	3 (Central Mudminnow)	1981-2017	https://lter.limnology.wisc.edu/data
Lake Kasumigaura (stations 3, 9)	Japan	36.027500	140.396310	167.7	7.4 [4]	Warm polymictic (ice-free)	3 (pond smelt)	1980-2018	http://db.cger.nies.go.jp/gem/moni-e/inter/GEMS/database/kasumi/index.html Matsuzaki, S.S., Suzuki, K., Kadoya, T., Nakagawa, M., & Takamura, N. (2018). Bottom-up linkages between primary production, zooplankton, and fish in a shallow, hypereutrophic lake. Ecology, 99(9), 2025-2036.
Yale Lake	FL, United States	28.912161	-81.735396	16.4		Warm polymictic (ice free)	4 (Largemouth Bass, Longnose Gar, Tilapia, Catfish, Grass Carp, many more)	1999-2016	St. Johns River Water Management District https://www.sjrwmd.com
Loch Leven	Scotland	56.194039	-3.375480	16.1	13 [4.6]	Dimictic (sporadic interannual ice cover)	3-4 (Brown trout)	1971-2007	data.gov.uk/ Gunn, I. D. M., George, D. G., Johnson, D., Jones, D. H., May, L. (2015). Crustacean zooplankton data from Loch Leven, 1972-2007. NERC Environmental Information Data Centre
Beauclair Lake	FL, United States	28.773431	-81.660099	4.6	[3.0]	Warm polymictic (ice free)	4 (Largemouth Bass, Black Crappies, Bowfin, many more)	1999-2016	St. Johns River Water Management District https://www.sjrwmd.com

Table S2. Zooplankton species from all datasets and their functional group classification.

Copepods	Small cladocerans	Large cladocerans	Predatory
Acanthocyclops vernalis	*Acroperus harpae*	*Daphnia ambigua*	*Bythotrephes longimanus*
Acanthocyclops sp.	*Alona cf quadrata*	*Daphnia carinata*	*Chaoborus* sp.
Aglaodiaptomus clavipes	*Alona costata*	*Daphnia cucullata*	*Leptodora kindti*
Boeckella propinqua	*Alona guttata*	*Daphnia dentifera*	*Polyphemus pediculus*
Canthocamptus staphylinus	*Alona karua*	*Daphnia dubia*	*Polyphemus* sp.
Cyclops abyssorum	*Alona* sp.	*Daphnia galeata*	
Cyclops bohater	*Bosmina fatalis*	*Daphnia hyalina*	
Cyclops strenuus	*Bosmina longirostris*	*Daphnia longiremis*	
Cyclops vicinus	*Bosmina meridionalis*	*Daphnia lumholtzi*	
Diacyclops thomasi	*Bosmina* sp.	*Daphnia mendotae*	
Diacyclops sp.	*Bosminopsis deitersi*	*Daphnia parvula*	
Diaptomus dorsalis	*Camptocercus* sp.	*Daphnia pulex*	
Diaptomus floridanus	*Ceriodaphnia cf dubia*	*Daphnia pulicaria*	
Eodiaptomus japonicus	*Ceriodaphnia cornuta*	*Daphnia retrocurva*	
Epischura lacustris	*Ceriodaphnia lacustris*	*Daphnia* sp.	
Ergasilus sp.	*Ceriodaphnia reticulata*	*Diaphanosoma birgei*	
Eucyclops agilis	*Ceriodaphnia rigaudi*	*Diaphanosoma brachyurum*	
Eucyclops elegans	*Ceriodaphnia* sp.	*Diaphanosoma* sp.	
Eucyclops serrulatus	*Chydorus sphaericus*	*Holopedium gibberum*	
Eucyclops sp.	*Chydorus* sp.	*Holopedium* sp.	
Eudiaptomus gracilis	*Disparalona rostrata*	*Ilyicryptus sordidus*	
Harpacticoid	*Eubosmina coregoni*	*Ilyocryptus spinifer*	
Leptodiaptomus minutus	*Eubosmina tubicen*	*Ophryoxus gracilis*	
Leptodiaptomus sicilis	*Kurzia latissima*		
Leptodiaptomus siciloides	*Leydigia leydigii*		
Megacyclops gigas	*Leydigia quadrangularis*		

Copepods	Small cladocerans	Large cladocerans	Predatory
Megacyclops viridis	*Leydigia* sp.		
Mesocyclops edax	*Macrothrix rosea*		
Mesocyclops sp.	*Moina macrocopa*		
Orthocyclops modestus	*Moina micrura*		
Paracyclops fibriatus	*Pleuroxus hamulatus*		
Paracyclops poppei	*Pleuroxus striatus*		
Pseudodiaptomus inopinus	*Scapholeberis* sp.		
Skistodiaptomus oregonensis	*Sinobosmina fryei*		
Skistodiaptomus pallidus			
Skistodiaptomus sp.			
Thermocyclops taihokuensis			
Tropocyclops prasinus prasinus			
Tropocyclops prasinusmexicanus			
Tropocyclops sp.			

Table S3. Proportion of variance explained (R^2), local weighting parameters (θ), and sample sizes (number of timepoints, n) for models of total zooplankton growth (bottom-up model), and chl-a growth (top-down model).

	**Bottom-up Model**			**Top-down Model**		
**Lake**	$\mathbf{R^2}$	$\boldsymbol{\theta}$	$\boldsymbol{n}$	$\mathbf{R^2}$	$\boldsymbol{\theta}$	$\boldsymbol{n}$
Lake Taupo	0.232	0.75	76	0.445	1.5	76
Crystal Lake (WI)	0.393	1	192	0.438	0.3	196
Sparkling Lake (WI)	0.444	1	147	0.699	1.5	159
Trout Lake (WI)	0.478	1.5	134	0.578	3	150
Big Muskellunge (WI)	0.362	1	131	0.681	0	147
Lake Geneva	0.469	2	417	0.558	2	422
Lake Zurich	0.479	2	211	0.380	1.5	211
Crystal Bog (WI)	0.568	1.5	120	0.304	0	134
Lake Mendota (WI)	0.435	0	91	0.580	2	91
Lake Kasumigaura	0.398	2	399	0.312	3	405
Yale Lake (FL)	0.330	0.5	96	0.329	3	97
Loch Leven	0.503	1.5	171	0.333	2	177
Beauclair Lake (FL)	0.303	1.5	169	0.249	1.5	174

Table S4. Analysis of variance tables for regression models for interaction strength coefficients across all lakes. The model for species richness is fit to the residuals from the first model. Predictors included as 2nd order orthogonal polynomials indicated by (poly).

	Bottom-Up Coefficients					Top-Down Coefficients				
**Predictor**	**Sum Sq**	**Df**	**F value**	**Pr(>F)**		**Sum Sq**	**Df**	**F value**	**Pr(>F)**	
Season	0.151	1	7.436	0.006	**	0.854	1	32.291	<0.001	***
Season_3	0.318	1	15.631	<0.001	***	0.002	1	0.091	0.763	
TSI (poly)	6.065	2	149.234	<0.001	***	9.978	2	188.604	<0.001	***
Temperature (poly)	1.136	2	27.947	<0.001	***	1.2	2	22.679	<0.001	***
Residuals	42.792	2106				55.706	2106			
Species Richness (poly)	2.452	2	60.077	<0.001	***	0.0826	2	2.276	0.103	
Residuals	33.024	1618				29.3538	1618			

Table S5. Analysis of variance tables for regression models for interaction strength coefficients within individual lakes for the effect of seasonality.

		Bottom-Up Coefficients					Top-Down Coefficients				
**Lake**	**Predictor**	**Sum Sq**	**Df**	**F value**	**Pr(>F)**		**Sum Sq**	**Df**	**F value**	**Pr(>F)**	
Lake Taupo	Season	0.78263	1	212.396	<0.001	***	0.51935	1	51.417	<0.001	***
	Season_3	0.25116	1	68.162	<0.001	***	0.06048	1	5.987	0.017	*
	Residuals	0.26899	73				0.73736	73			
Crystal Lake (WI)	Season	0.05754	1	57.701	<0.001	***	6.6E-05	1	1.844	0.177	
	Season_3	0.02651	1	26.578	<0.001	***	8.2E-05	1	2.296	0.132	
	Residuals	0.12067	121				0.00431	121			
Sparkling Lake (WI)	Season	0.15262	1	121.686	<0.001	***	0.02119	1	27.916	<0.001	***
	Season_3	0.02652	1	21.148	<0.001	***	0.0004	1	0.528	0.469	
	Residuals	0.11413	91				0.06908	91			
Trout Lake (WI)	Season	0.61462	1	47.163	<0.001	***	0.16621	1	9.054	0.003	.
	Season_3	0.21505	1	16.502	<0.001	***	0.27309	1	14.876	<0.001	***
	Residuals	1.18588	91				1.67051	91			
Big Muskellunge (WI)	Season	0.3448	1	81.034	<0.001	***	3.8E-06	1	0.889	0.348	
	Season_3	0.00374	1	0.878	0.351		5.8E-06	1	1.370	0.245	
	Residuals	0.34466	81				0.00035	81			
Lake Geneva	Season	1.94898	1	268.600	<0.001	***	0.40827	1	119.133	<0.001	***
	Season_3	0.16494	1	22.732	<0.001	***	0.28048	1	81.844	<0.001	***
	Residuals	2.86614	395				1.35365	395			
Lake Zurich	Season	0.51921	1	162.945	<0.001	***	0.10495	1	34.082	<0.001	***
	Season_3	0.00212	1	0.665	0.416		0.04215	1	13.688	<0.001	***
	Residuals	0.54806	172				0.52964	172			

		Bottom-Up Coefficients					Top-Down Coefficients				
**Lake**	**Predictor**	**Sum Sq**	**Df**	**F value**	**Pr(>F)**		**Sum Sq**	**Df**	**F value**	**Pr(>F)**	
Crystal Bog (WI)	Season	0.01776	1	8.504	0.005		4.4E-06	1	0.095	0.759	
	Season_3	0.06311	1	30.213	<0.001	***	1.5E-06	1	0.032	0.858	
	Residuals	0.17128	82				0.00382	82			
Lake Mendota (WI)	Season	1.5E-06	1	0.019	0.890		6.8E-05	1	0.004	0.950	***
	Season_3	6.8E-05	1	0.872	0.353		0.99138	1	57.268	<0.001	***
	Residuals	0.00671	86				1.48877	86			
Lake Kasumigaura	Season	0.2995	1	60.305	<0.001	***	0.04534	1	1.345	0.247	
	Season_3	0.05018	1	10.104	0.002	**	0.25728	1	7.632	0.006	**
	Residuals	1.96172	395				13.3151	395			
Yale Lake (FL)	Season	0.01588	1	10.648	0.002	**	0.22698	1	1.230	0.270	
	Season_3	0.03258	1	21.839	<0.001	***	0.71975	1	3.900	0.051	.
	Residuals	0.13873	93				17.1652	93			
Loch Leven	Season	3.5E-05	1	0.016	0.900		3.01534	1	221.911	<0.001	***
	Season_3	0.01289	1	5.754	0.018	*	0.64743	1	47.647	<0.001	***
	Residuals	0.34284	153				2.07898	153			
Beauclair Lake (FL)	Season	2.06656	1	297.846	<0.001	***	0.45476	1	126.005	<0.001	***
	Season_3	1.20636	1	173.868	<0.001	***	0.06673	1	18.488	<0.001	***
	Residuals	1.14483	165				0.5955	165			

Table S6. Analysis of variance tables for regression models for interaction strength coefficients within individual lakes. Predictors were included as 2nd order orthogonal polynomials.

		Bottom-Up Coefficients					Top-Down Coefficients				
**Lake**	**Predictor (poly)**	**Sum Sq**	**Df**	**F value**	**Pr(>F)**		**Sum Sq**	**Df**	**F value**	**Pr(>F)**	
Lake Taupo	TSI	0.06463	2	6.834	0.002	**	0.01916	2	0.922	0.403	
	Temperature	0.11114	2	11.752	<0.001	***	0.156	2	7.508	0.001	**
	Zooplankton Abundance	0.04043	2	4.275	0.018	***	0.03444	2	1.658	0.198	
	Species Richness	0.03881	2	4.103	0.021	*	0.03632	2	1.748	0.182	
	Residuals	0.31683	67				0.69603	67			
Crystal Lake (WI)	TSI	0.01881	2	9.887	<0.001	***	9.3E-05	2	1.514	0.224	
	Temperature	0.02021	2	10.622	<0.001	***	0.00022	2	3.581	0.031	*
	Zooplankton Abundance	0.00193	2	1.015	0.366		0.00065	2	10.565	<0.001	***
	Species Richness	0.00321	2	1.685	0.190		0.00013	2	2.070	0.131	
	Residuals	0.10939	115				0.00354	115			

		Bottom-Up Coefficients					Top-Down Coefficients				
**Lake**	**Predictor (poly)**	**Sum Sq**	**Df**	**F value**	**Pr(>F)**		**Sum Sq**	**Df**	**F value**	**Pr(>F)**	
Sparkling	TSI	0.00936	2	1.396	0.253		0.00214	2	1.284	0.282	
Lake (WI)	Temperature	0.0023	2	0.344	0.710		0.00229	2	1.379	0.257	
	Zooplankton Abundance	0.03219	2	4.801	0.011	*	0.00663	2	3.988	0.022	*
	Species Richness	0.01213	2	1.810	0.170		0.00212	2	1.274	0.285	
	Residuals	0.28494	85				0.0707	85			
Trout Lake	TSI	0.26484	2	10.895	<0.001	***	0.00925	2	0.237	0.789	
(WI)	Temperature	0.17093	2	7.032	0.001	**	0.11721	2	3.008	0.055	.
	Zooplankton Abundance	0.02678	2	1.102	0.337		0.05666	2	1.454	0.239	
	Species Richness	0.02758	2	1.135	0.326		0.03579	2	0.918	0.403	
	Residuals	1.03309	85				1.65614	85			
Big	TSI	0.07018	2	5.133	0.008	**	2.2E-06	2	0.251	0.779	
Muskellunge	Temperature	0.05678	2	4.153	0.019	*	1.7E-05	2	1.919	0.154	
(WI)	Zooplankton Abundance	0.00527	2	0.385	0.682		1.4E-06	2	0.164	0.849	
	Species Richness	0.04533	2	3.315	0.042	*	2.5E-06	2	0.281	0.756	
	Residuals	0.51269	75				0.00033	75			
Lake Geneva	TSI	0.0086	2	0.580	0.560	.	0.00152	2	0.179	0.836	
	Temperature	0.84326	2	56.902	<0.001	***	0.04045	2	4.775	0.009	**
	Zooplankton Abundance	0.41594	2	28.067	<0.001	***	0.36404	2	42.978	<0.001	***
	Species Richness	0.01637	2	1.104	0.332		0.02133	2	2.519	0.082	.
	Residuals	2.88239	389				1.64745	389			
Lake Zurich	TSI	0.00671	2	0.718	0.489		0.00109	2	0.189	0.828	
	Temperature	0.21972	2	23.492	<0.001	***	0.06214	2	10.823	<0.001	***
	Zooplankton Abundance	0.0024	2	0.257	0.774		0.00658	2	1.145	0.321	
	Species Richness	0.01709	2	1.827	0.164		0.02072	2	3.609	0.029	*
	Residuals	0.7763	166				0.47651	166			
Crystal Bog	TSI	0.00115	2	0.224	0.800		0.0002	2	2.357	0.102	
(WI)	Temperature	0.02373	2	4.613	0.013	*	0.0001	2	1.212	0.303	
	Zooplankton Abundance	0.00039	2	0.075	0.928		0.00016	2	1.886	0.159	
	Species Richness	0.00312	2	0.607	0.547		0.00016	2	1.880	0.160	
	Residuals	0.19545	76				0.00324	76			

		Bottom-Up Coefficients					Top-Down Coefficients				
**Lake**	**Predictor (poly)**	**Sum Sq**	**Df**	**F value**	**Pr(>F)**		**Sum Sq**	**Df**	**F value**	**Pr(>F)**	
Lake Mendota (WI)	TSI	0.00022	2	1.420	0.248		0.00721	2	0.126	0.882	
	Temperature	9.7E-05	2	0.635	0.533		0.17456	2	3.058	0.053	.
	Zooplankton Abundance	0.00029	2	1.881	0.159		0.12787	2	2.240	0.113	
	Species Richness	0.00015	2	0.969	0.384		0.01329	2	0.233	0.793	
	Residuals	0.00613	80				2.2833	80			
Lake Kasumigaura	TSI	0.02922	2	3.009	0.050		0.41724	2	6.478	0.002	***
	Temperature	0.09327	2	9.604	<0.001	**	0.03045	2	0.473	0.624	
	Zooplankton Abundance	0.18387	2	18.933	<0.001	***	0.26427	2	4.103	0.017	*
	Species Richness	0.21203	2	21.833	<0.001	***	0.0178	2	0.276	0.759	
	Residuals	1.88892	389				12.5269	389			
Yale Lake (FL)	TSI	0.02276	2	9.223	<0.001	***	3.80391	2	12.948	<0.001	***
	Temperature	0.01938	2	7.851	0.001	***	0.65966	2	2.245	0.112	
	Zooplankton Abundance	0.00684	2	2.772	0.068	.	0.89248	2	3.038	0.053	.
	Residuals	0.10982	89				13.0731	89			
Loch Leven	TSI	0.01377	2	3.202	0.043	*	2.0811	2	58.405	<0.001	***
	Temperature	0.00099	2	0.229	0.795		1.31074	2	36.785	<0.001	***
	Zooplankton Abundance	0.01688	2	3.927	0.022	*	0.01529	2	0.429	0.652	
	Residuals	0.32032	149				2.65462	149			
Beauclair Lake (FL)	TSI	0.04231	2	0.839	0.434		0.11792	2	11.050	<0.001	***
	Temperature	0.25545	2	5.068	0.007	.	0.07417	2	6.950	0.001	*
	Zooplankton Abundance	0.18481	2	3.666	0.028	*	0.06933	2	6.496	0.002	**
	Residuals	4.05776	161				0.85907	161			

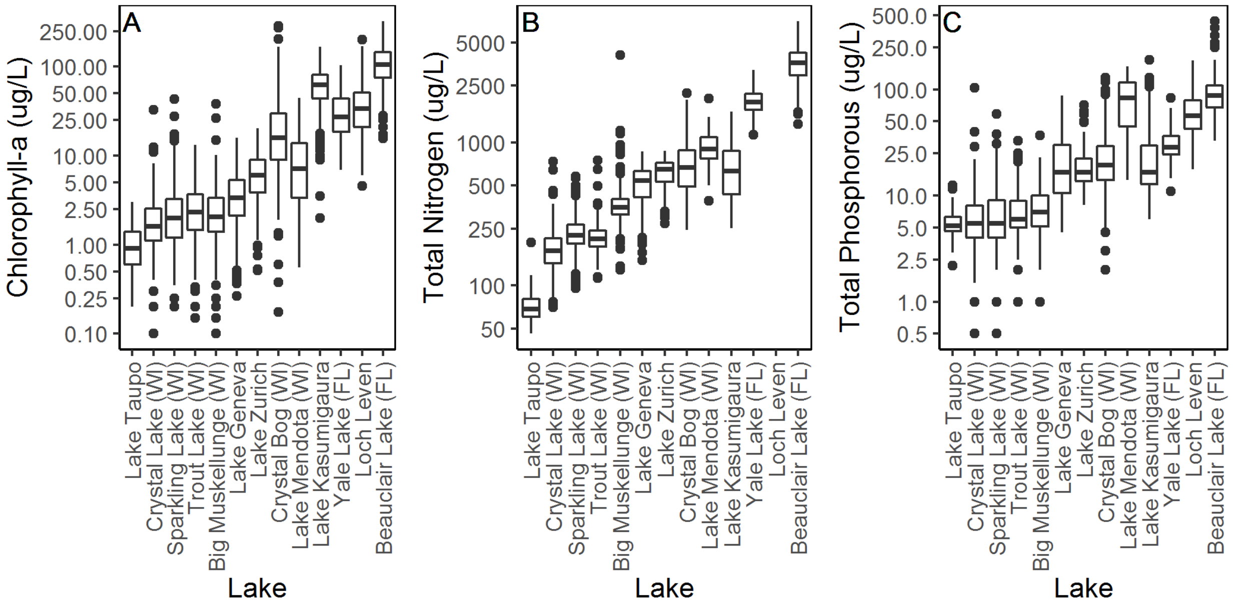

Fig. S1. Chlorophyll-a (A), total nitrogen (B), and total phosphorus (C) concentrations in 13 lakes. Note that all values are plotted on log scales.

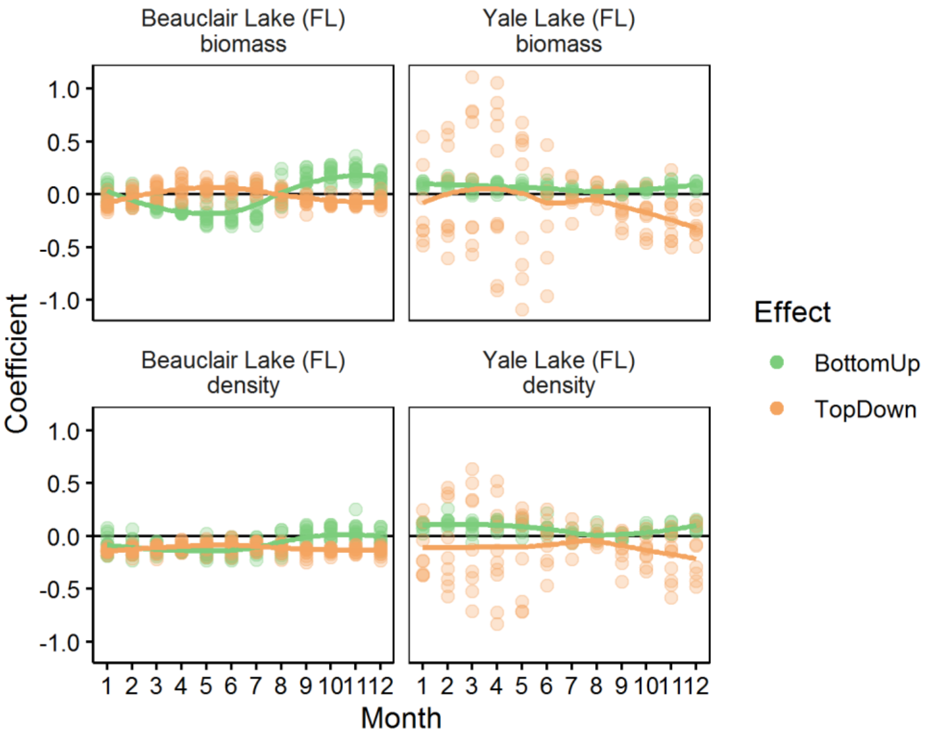

Fig. S2. Seasonal variation in BU and TD interaction strength coefficients within lakes using either both zooplankton biomass or density. Curves are splines. The BU and TD species interaction strengths modelled with biomass and density are highly correlated in both lakes at r≈0.83.

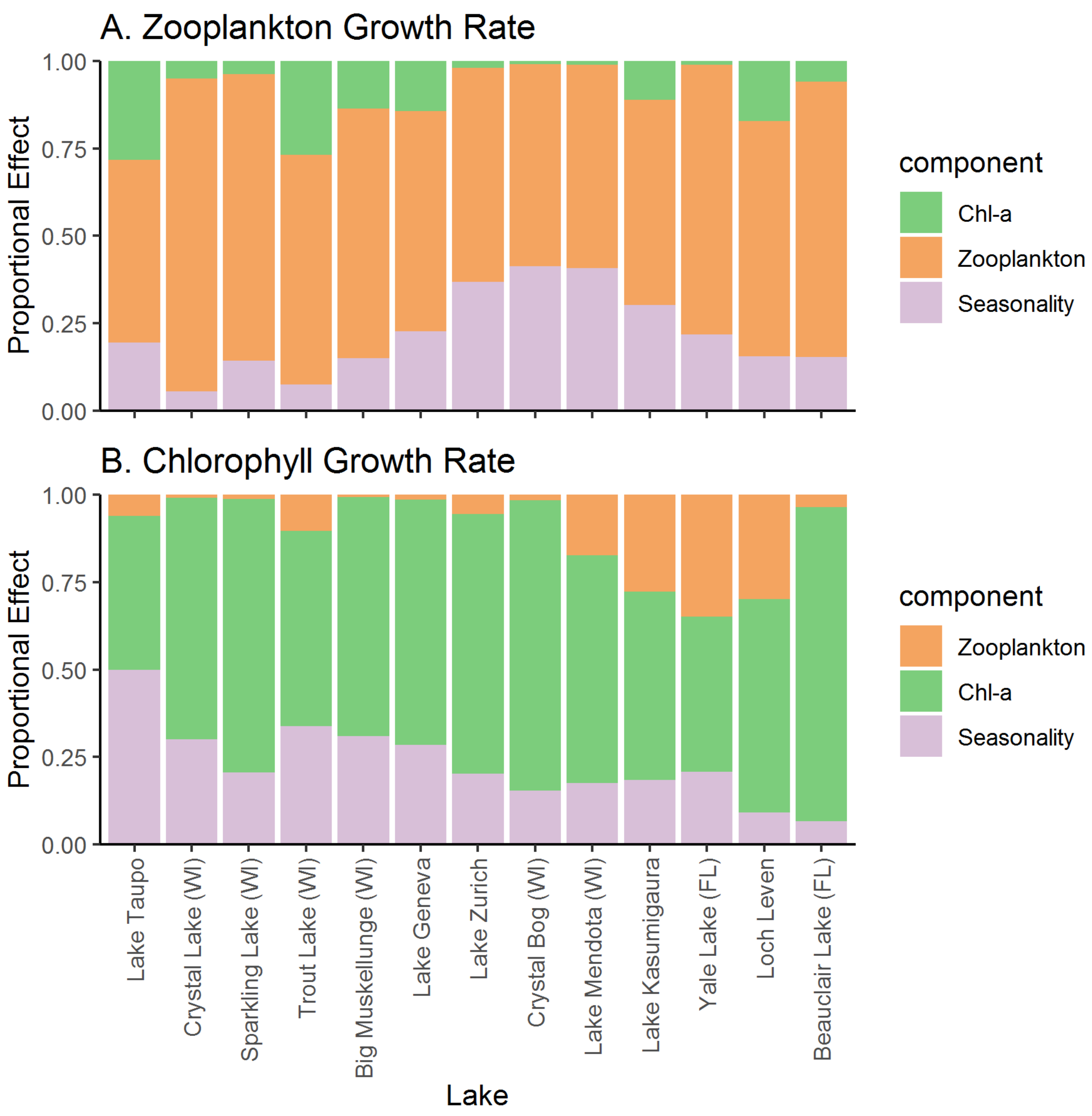

Fig. S3. Proportional effects of chl-a, zooplankton, and seasonality on the growth rates of (A) zooplankton and (B) chl-a in 13 lakes. Values reflect the proportion of the explained variance in growth rate attributable to each set of predictors.

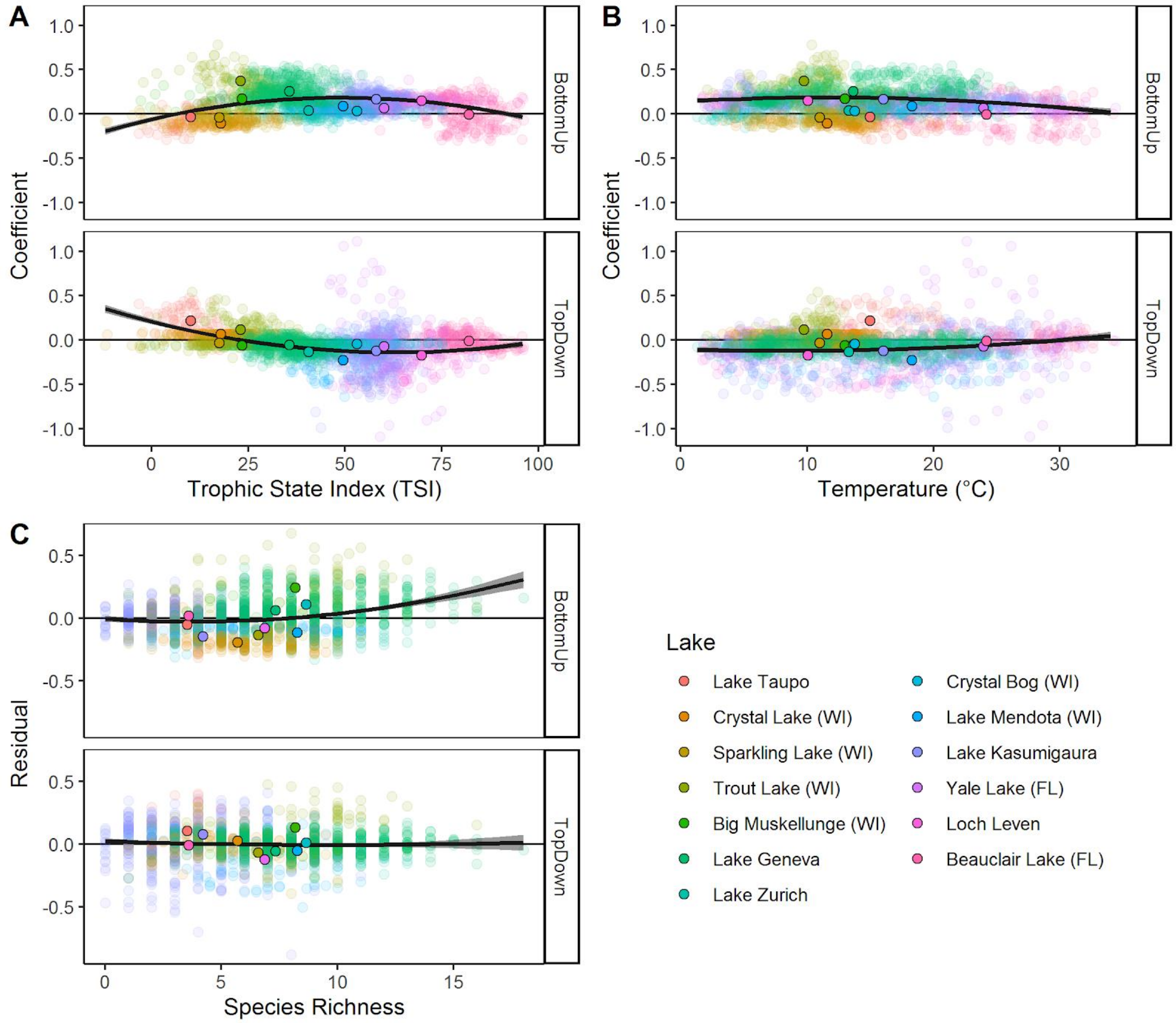

Fig. S4. Conditional effects of (A) TSI, (B) temperature, and (C) zooplankton species richness on BU and TD interaction strength for herbivorous zooplankton, evaluated across lakes. Models are fit to all data points shown. For reference, points with dark outlines indicate the means for each lake. Effect of species richness is evaluated after accounting for the effect of TSI and temperature (using model residuals, see text).

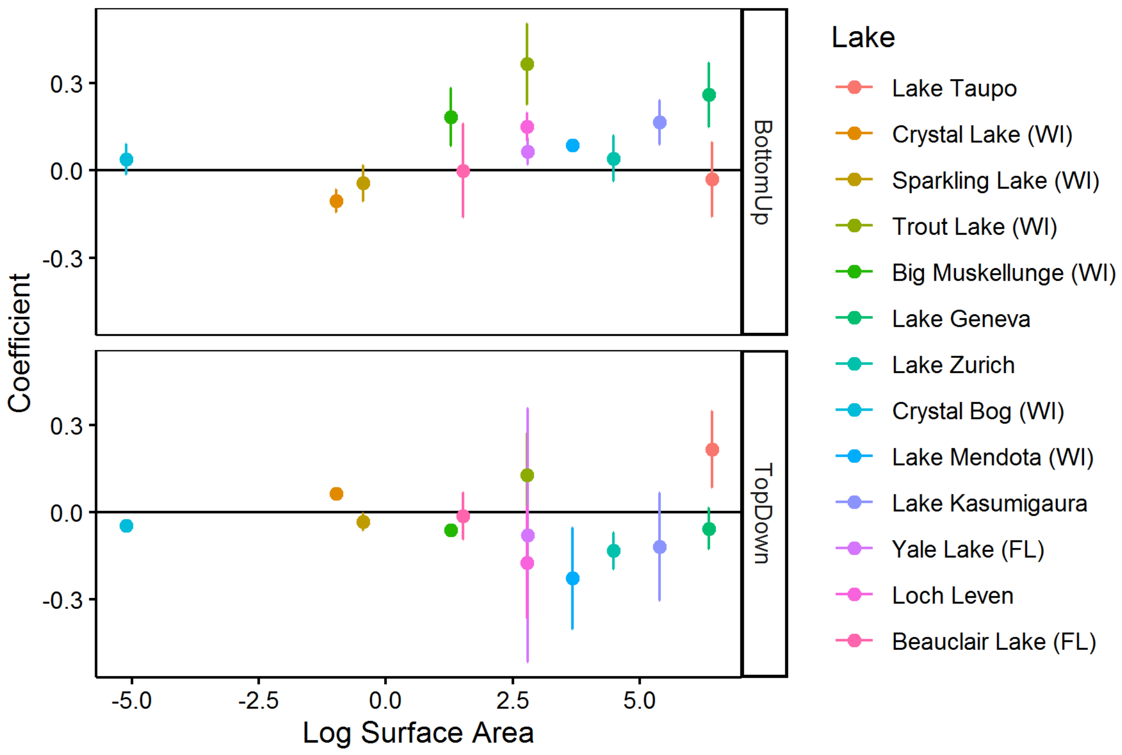

Fig. S5. The relationship between lake surface area and BU and TD interaction strength after accounting for the effect of TSI and temperature. Lake size (natural log surface area) was not correlated with mean TSI (r = -0.04).

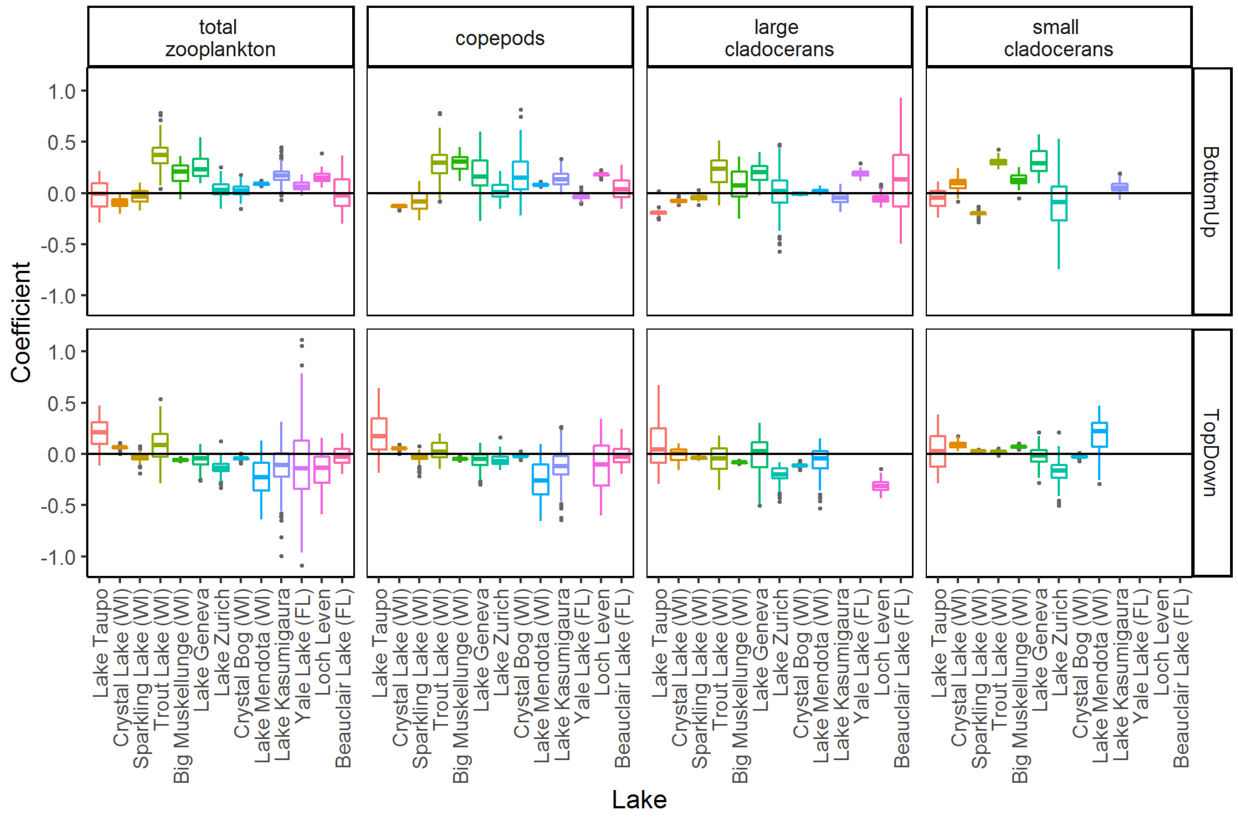

Fig. S6. Distribution of BU and TD coefficients for total herbivorous zooplankton and different herbivorous zooplankton functional groups in 13 lakes. Where boxplots are not shown, the functional group was either not observed enough to analyze (time series was > 60% zeros), or the fitted model had an R^2 value < 0.2.
